# Enhanced functionalities for annotating and indexing clinical text with the NCBO Annotator+

**DOI:** 10.1093/bioinformatics/bty009

**Published:** 2018-01-12

**Authors:** Andon Tchechmedjiev, Amine Abdaoui, Vincent Emonet, Soumia Melzi, Jitendra Jonnagaddala, Clement Jonquet

**Affiliations:** 1 Laboratory of Informatics, Robotics and Microelectronics of Montpellier (LIRMM), University of Montpellier & CNRS, Montpellier, France; 2 Faculty of Medicine, University of New South Wales, Sydney, New South Wales, Australia; 3 Center for Biomedical Informatics Research (BMIR), Stanford University, Stanford, California, USA

## Abstract

**Summary:**

Second use of clinical data commonly involves annotating biomedical text with terminologies and ontologies. The National Center for Biomedical Ontology Annotator is a frequently used annotation service, originally designed for biomedical data, but not very suitable for clinical text annotation. In order to add new functionalities to the NCBO Annotator without hosting or modifying the original Web service, we have designed a proxy architecture that enables seamless extensions by pre-processing of the input text and parameters, and post processing of the annotations. We have then implemented enhanced functionalities for annotating and indexing free text such as: scoring, detection of context (negation, experiencer, temporality), new output formats and coarse-grained concept recognition (with UMLS Semantic Groups). In this paper, we present the NCBO Annotator+, a Web service which incorporates these new functionalities as well as a small set of evaluation results for concept recognition and clinical context detection on two standard evaluation tasks (Clef eHealth 2017, SemEval 2014).

**Availability and implementation:**

The Annotator+ has been successfully integrated into the SIFR BioPortal platform—an implementation of NCBO BioPortal for French biomedical terminologies and ontologies—to annotate English text. A Web user interface is available for testing and ontology selection (http://bioportal.lirmm.fr/ncbo_annotatorplus); however the Annotator+ is meant to be used through the Web service application programming interface (http://services.bioportal.lirmm.fr/ncbo_annotatorplus). The code is openly available, and we also provide a Docker packaging to enable easy local deployment to process sensitive (e.g. clinical) data in-house (https://github.com/sifrproject).

**Supplementary information:**

Supplementary data are available at *Bioinformatics* online.

## 1 Introduction

Semantic annotation of clinical data with standard medical terminologies/ontologies facilitates second use and translational data discoveries. Electronic Health Records often include unstructured elements (free text) that contain valuable information for medical research (Meystre *et al.*, 2008). Researchers have developed systems to automatically detect clinical conditions and extract valuable knowledge in order to facilitate decision support (Rothman *et al.*, 2012), the identification of patients (Liu *et al.*, 2013) and surveillance (Herasevich *et al.*, 2011). In 2009, the US National Center for Biomedical Ontologies released the NCBO Annotator (Jonquet *et al.*, 2009) within the BioPortal platform (Noy *et al.*, 2009), a publicly accessible and easily usable annotator Web service to process raw biomedical English text and identify ontology concepts. The annotation workflow is based on a highly efficient syntactic concept recognition tool [95% precision for diseases (Dai *et al.*, 2008)] that uses concept names and synonyms. The recognizer optionally allows to use names and synonyms of related concepts through semantic expansion [e.g. *is_a* assertions and concept-to-concept mappings (Shah *et al.*, 2009)]. The NCBO Annotator has been widely adopted in the community and is one of the most actively used services from NCBO BioPortal, with a dictionary made from labels of 600+ ontologies. Yet, the Annotator lacks natural language processing capabilities (e.g. handling of morphological variants, disambiguation) required to improve the accuracy of annotations. Another limitation is the absence of scoring and of the contextualization of clinical text annotations, something it was never really designed for.

In the context of the Semantic Indexing of French biomedical Resources (SIFR) project, in which we have developed a French version of the Annotator, we have implemented some new features for French that we seamlessly ported to English through a proxy Web service called NCBO Annotator+. These new features include: annotation scoring, additional output formats (for evaluation and integration with standard clinical systems), clinical context detection (negation, experiencer and temporality through the integration of the NegEx/ConText algorithm) and coarse-grained entity type annotations (with UMLS Semantic Groups, e.g. anatomy, disorders, devices). This article presents: (i) the proxy architecture and on how it enables the addition of new features, (ii) a performance evaluation of the NCBO Annotator+ on concept recognition tasks (death certificates and clinical notes) and on context detection (clinical notes only).

## 2 Materials and methods

**Fig. 1.**
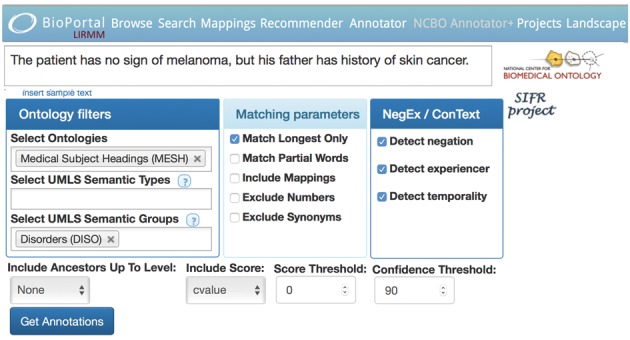
User Interface of the NCBO Annotator+ Web service (http://bioportal.lirmm.fr/ncbo_annotatorplus) illustrating new features. To reproduce this example with the Web service, use the URL: https://goo.gl/BTrNzJ

**Fig. 2.**
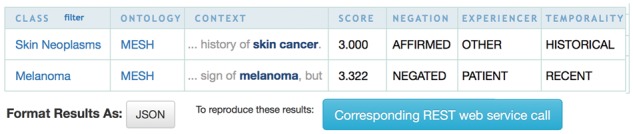
Annotation results for the example sentence from Figure 1

Annotator+ is composed of a Web user interface in the SIFR BioPortal, and a proxy servlet to implement new features; it uses the NCBO BioPortal Annotator REST API in the backend. Figure 1 illustrates the Annotator+ interface with an example sentence (Restricted to the MESH and SNOWMED-CT vocabularies, filtered on the ‘Disorder’ UMLS Semantic Group, scored with a 90% relative threshold and with clinical context detection activated), while Figure 2 illustrates the resulting annotations.

### 2.1 Proxy Web service architecture

**Fig. 3.**
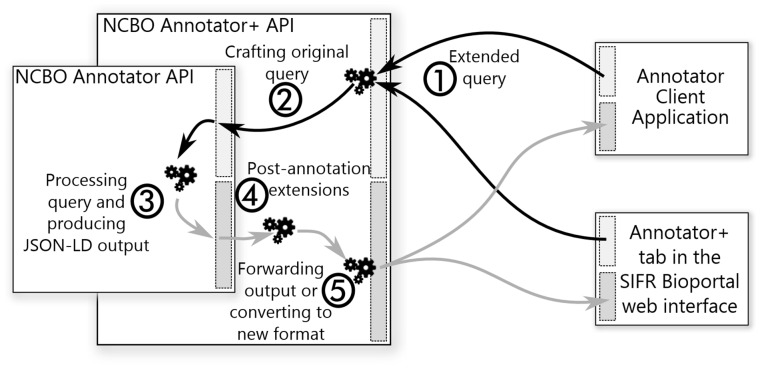
NCBO Annotator+ proxy-like Web service architecture

The NCBO Annotator is developed and maintained by the NCBO and does not easily support quick add-ons. To extend the NCBO Annotator without modifying the original application, we developed a proxy Web service architecture that can run independently and extend the service by pre-processing inputs and post-processing outputs. It works as follows (Fig. 3): (i) requests are sent to the proxy with extended parameters that are parsed to select/apply the additional features; (ii) a query is crafted for the original service without any extended parameters; (iii) the original NCBO Annotator processes the query and returns the results; (iv) the proxy retrieves annotations and applies post-processing/filtering (e.g. scoring); and finally, (v) the output is generated in the original format or in one of the new output formats from Annotator+. The proxy is implemented in a generic form that enables the querying of any NCBO-like annotator Web service. Indeed, we also use it for the French Annotator (Jonquet *et al.*, 2016a,b) and the AgroPortal Annotator, a similar Web service developed for agronomy (Jonquet *et al.*, 2016a).

### 2.2 New features

**Scoring.** During semantic indexing, annotations ‘bring together’ data elements and ontology concepts. Annotation scoring and ranking help to distinguish the most relevant annotations for a given element (e.g. a document, a clinical report) and when searching the original data. Typically, in information retrieval approaches, scoring is based on term frequency. We have implemented and evaluated a new scoring method for that purpose. By using a natural language processing term extraction measure called C-Value (Frantzi *et al.*, 2000), we were able to offer three scoring algorithms based on match frequencies that favour longer multi-word term annotations (higher scores) over shorter or single word annotation (Melzi *et al.*, 2014). We also added a mechanism to filter annotations by absolute score or in proportion (percentage) to the cumulative score distribution, to retrieve only the most relevant annotations (e.g. annotating with a threshold of 90% only retains the annotations with scores in the top 10% of the score distribution).

**New output formats.** NCBO Annotator supports XML and JSON-LD outputs. While JSON-LD is a recognized format, it is not sufficient for many annotation benchmarks and tasks, especially in the semantic Web and natural-language-processing communities. Annotator+ adds support for standard (BRAT, RDF) and task-specific (e.g. CLEF eHealth) formats. RDF is the backbone language of the semantic Web and BRAT (http://brat.nlplab.org) is widely used for evaluation campaigns and for the production of annotated corpora. We also enriched the JSON-LD output with additional information (e.g. scores or clinical context).

**Clinical context.** For clinical text, the context of the annotated clinical conditions is crucial: Distinguishing between affirmed and negated occurrences (e.g. ‘no sign of metastasis’); whether a condition pertains to the patient or to others (e.g. ‘mother had breast cancer’); or temporality (i.e. if a condition is recent or historical. e.g. ‘history of poliovirus’). NegEx/ConText, is one of the best performing and fastest (open-source) algorithms for clinical context detection in English medical text (Harkema *et al.*, 2009). NegEx/ConText is based on lexical cues (trigger terms) that modify the default status of medical conditions appearing in their scope. For instance, by default the system considers a condition *affirmed*, and marks it as *negated* only if it appears under the scope of a trigger term. Each trigger term has a pre-defined scope either forward (e.g. ‘denies’) or backward (e.g. ‘is ruled out’), which ends by a colon or a termination term (e.g. ‘but’). We integrated this algorithm within the NCBO Annotator+ by post-processing the sentence in which an annotation appears. To our knowledge, this is the first implementation of a Web-based ConText-like system in a publicly accessible platform allowing non-experts in natural-language-processing to both annotate and contextualize medical conditions in clinical notes.

**Coarse-grained semantic annotation.** Recognizing broad entity types (e.g. gene, drug, disease) is a task of high interest for the BioNLP community. The 10 Semantic Groups (McCray *et al.*, 2001) are often used as coarse-grained groupings of the Unified Medical Language System (UMLS) Semantic Types (Bodenreider, 2004). Thanks to the capability of the NCBO Annotator to filter ontologies by Semantic Types, we have also added the capability to filter by Semantic Groups in Annotator+. This enables anyone to annotate free text and keep only certain broad types of annotations. For instance, a pharmacogenomics researcher doing a study may restrict the annotations to the types ‘disorders’ and ‘chemicals & drugs’ to investigate the effect of adverse drug reactions.

### 2.3 Evaluation protocol

We briefly report on the performance of the NCBO Annotator+ for: (i) annotating and contextualizing concepts in clinical text on the CLEF eHealth 2017 task 1 corpus (Névéol *et al.*, 2017), created for the automatic annotation of death certificates with ICD-10 codes; (ii) the SemEval 2015 Task 14.2 development corpus, created for the identification of biomedical concepts (i.e. names and identifiers in UMLS) and of clinical context features (we covered negation and experiencer).

## 3 Results and discussion

This section provides: (i) benchmark results for concept recognition with the original NCBO Annotator and (ii) evaluation of the new features (negation & experiencer detection only) of the Annotator+. The goal is both to provide additional performance evaluations to the community of the NCBO Annotator and to evaluate our own additions to the Annotator+. In 2017, we have participated to the CLEF eHealth 2017 Task 1 evaluation campaign, with the French/SIFR Annotator and the NCBO Annotator+. The campaign tackles the problem of information extraction (diagnostic coding) in written death certificates, where the objective is to annotate each document with a set of relevant International Classification of Diseases, 10th revision (ICD-10) diagnostic codes. We have built a custom SKOS vocabulary (Simple Knowledge Organization System) from the dictionary of terms provided and uploaded it to the NCBO BioPortal (which also parses SKOS as input format). When annotating the death certificates with the NCBO Annotator, we obtained median results compared to the rest of the competitors [cf. Table 1 (Névéol *et al.*, 2017; Tchechmedjiev *et al.*, 2017)]; ahead of other knowledge-based systems but behind specifically tailored supervised learning systems. The results are encouraging considering that we have not customized the service in any way for the task. We acknowledge the better performance of supervised learning approaches, but claim that in the health domain, they are often not applicable for lack of training data.

**Table 1.** Evaluation for concept recognition (NCBO Annotator) and clinical context detection (Annotator+) expressed by Precision, Recall, F-measure, Accuracy)

Task (Corpus)	P (%)	R (%)	F1 (%)	A (%)
Concept Recognition (CLEF eHealth)	69.1	51.4	58.9	
Concept Recognition (SemEval)	46.9	62.0	53.4	66.6
Negation Detection (SemEval)	87.0	88.9	88.0	89.3
Experiencer Detection (SemEval)	52.9	70.4	60.4	52.7

For the evaluation of our integration of NegEx/ConText within the Annotator+, we used the SemEval 2015 corpus. For the task of concept recognition in the SemEval corpus, the NCBO Annotator obtained average scores, given that we performed no adaptation to the task (and we did not use the training data at all), the concept recognition accuracy is fair (66.6%). We did not have access to the test gold standard and thus cannot compare to other participants (we ran on the dev. corpus). For negation, Annotator+ obtained state-of-the-art performance (balanced weighted average performance) and for experiencer detection, we obtain results that are not substantially lower than existing evaluations of ConText (Harkema *et al.*, 2009). These results confirm both the potential of the NCBO Annotator as a concept recognition service (never evaluated on standardized evaluation campaign tasks) and the nonreduced performance of NegEx/ConText when implemented in Annotator+.

## 4 Conclusion

We believe the NCBO Annotator+ offers a valuable framework to: (i) leverage an already performant service, which uses the biggest biomedical terms dictionary (600+ semantic resources including almost all UMLS and all the OBO Library ontologies); and (ii) improve the performance of this service on specific types of text such as in our case clinical notes. In the future, we will work on two important weaknesses of the service: disambiguation of annotations (too many polysemic terms decrease precision) and for clinical text mainly, cleaning and reformatting of the text (abbreviations, spelling mistakes, unconventional sentence structures, decrease recall). We working with the NCBO towards integrating some of this work directly into the NCBO Annotator.

## Supplementary Material

Supplementary Data
