# Supplementary material for: Enhanced functionalities for annotating and indexing clinical text with the NCBO Annotator+
Source: Bioinformatics. 2018 Jan 12;34(11):1962–5. doi: 10.1093/bioinformatics/bty009 (PMC5972606; doi:10.1093/bioinformatics/bty009)
Supplement: Supplementary Data [file bty009_supplement_bioinf-2017-1427.r2-3.pdf]

Enhanced Functionalities for Annotating and Indexing Clinical Text with the NCBO Annotator+

Andon Tchechmedjiev,[1,*] Amine Abdaoui,[1] Vincent Emonet,[1] Soumia Melzi,[1] Jitendra Jonnagaddala[2] and Clement Jonquet[1,3]

[1] Laboratory of Informatics, Robotics and Microelectronics of Montpellier (LIRMM), University of Montpellier & CNRS, France.
[2] Faculty of Medicine, University of New South Wales, Sydney, Australia.
[3] Center for Biomedical Informatics Research (BMIR), Stanford University, USA.

Supplementary material. Bioinformatics Submission BIOINF-2017-1427.R2.

Support for NCBO Annotator+ and the SIFR French Annotator: sifrportal-support@lirmm.fr
Documentation index: https://github.com/sifrproject/bioportal_web_ui/wiki

I. Proxy Architecture

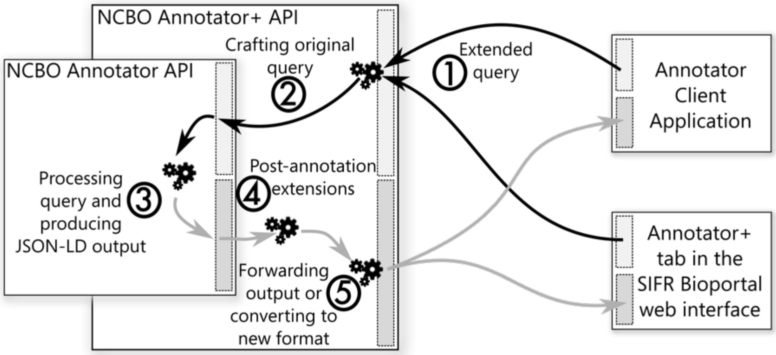

Figure 1. NCBO Annotator+ proxy-like Web service architecture

The NCBO Annotator+ architecture follows the proxy architectural pattern. We have created a front-end REST API that is to be queried instead of NCBO Annotator API. This service supports all the API functions supported by NCBO Annotator. Within the confines of the API of the original NCBO Annotator, our service merely forwards the request to NCBO Annotator and returns result as-is. If a user queries the NCBO Annotator+ API with an extended parameter, then the parameter is stripped and appropriate pre-processing steps are applied, before crafting a query to the original annotator and potentially post processing the annotation results.

The proxy architecture has been applied to produce NCBO Annotator+, however it is generic and is also used on the French Annotator in the SIFR BioPortal (http://bioportal.lirmm.fr/annotator) or on the AgroPortal Annotator (http://agroportal.lirmm.fr/annotator).

II. Application Programming Interface Guide

We are fully compatible with the original NCBO Annotator API, which is described in detail on the NCBO website: http://data.bioontology.org/documentation#nav_annotator. We will describe all the new parameters added by our proxy in the table below:

Parameter	Description
`semantic_groups=GROUP1,GROUP2,...`	Filter the annotations by one or more UMLS Semantic Groups as defined in: https://www.ncbi.nlm.nih.gov/pmc/articles/PMC4300099/
`format=[json|brat|quaero|rdf]`	Selects the output format of the annotations among json-ld, brat, quaero (a custom BRAT format for the Quaero annotated corpus), and rdf (a custom RDF format using the Annoation Ontology).
`score=[old|cvalue|cvalueh]`	Activate annotations scoring, takes a value among: `old, cvalue` (the c-value score as described in the paper) and cvalueh (a hierarchical version of c-value). The score is added to the annotatedClass of the JSON output as `score`.
`score_threshold=[0-9]+`	Filters the annotation by an absolute score threshold. Only annotations with a score above the threshold will be shown. Requires `score` to be activated.
`confindence_threshold=0-100`	Filters the annotations by a threshold between 0% and 100% relative to the distribution of scores in the output annotations. For example, a value of 90% will only keep annotations with scores in the top 90% of the score distribution.
`negation=[true|false]`	Activate negation detection with the ConText algorithm. The output is added to the annotations object of the JSON output as `negationContext`.
`experiencer=[true|false]`	Activate experiencer detection with the ConText akgorithm. The output is added to the annotations object of the JSON output as `experiencerContext`.
`temporality=[true|false]`	Activate temporality detection with the ConText algorithm. The output is added to the annotations object of the JSON output as `temporalityContext`.

Here's an example that illustrates most of the features:

http://services.bioportal.lirmm.fr/ncbo_annotatorplus/?
text=The patient has no sign of melanoma but his father had skin cancer.
&**ontologies**=MESH
&**longest_only**=true&**exclude_numbers**=false&**whole_word_only**=true&**exclude_synonyms**=false&
expand_mappings=false
&**negation**=true&**experiencer**=true&**temporality**=true
&**score**=cvalue
&**semantic_groups**=DISO
&**display_links**=false&**display_context**=false
&**apikey**=4a5011ea-75fa-4be6-8e89-f45c8c84844e

For simplicity use the following link: https://goo.gl/BTrNzJ

Here is an example of the JSON-LD output illustrating how the new features affect the output in practice:


```
[{...}, {
  "annotatedClass": {
    "@id": "http://purl.bioontology.org/ontology/MESH/D008545",
    "@type": "http://www.w3.org/2002/07/owl#Class"
  },
  "hierarchy": [],
  "annotations": [
    {
      "from": 28,
      "to": 35,
```

```
      "matchType": "PREF",
      "text": "MELANOMA",
      "negationContext": "NEGATED",
      "temporalityContext": "RECENT",
      "experiencerContext": "PATIENT"
    }
  ],
  "mappings": [],
  "score": 3.3219280948873626
}]
```


To illustrate the genericity of the architecture, we also give an example for French text on the SIFR Annotator with a BRAT output: https://goo.gl/4VtPpL


```
T1      Diso 30 38      mélanome
#1      AnnotatorNotes T1       http://purl.lirmm.fr/ontology/MSHFRE/D008545
A1      Negation-Negated T1
T2      Diso 81 98      cancer de la peau
#2      AnnotatorNotes T2       http://purl.lirmm.fr/ontology/MSHFRE/D012878
A2      Experiencer-Other T2
```


Please note that the apikeys provided in the example are merely demo apikeys. Should one wish to use the service more intensively, an account must be created (On NCBO BioPortal for Annotator+ and on SIFR BioPortal for the French service).
